# Developing the Community Paramedicine Needs Assessment Tool

**DOI:** 10.3390/nursrep15120440

**Published:** 2025-12-10

**Authors:** Tyne M. Markides, Brendan Shannon, Cheryl Cameron, Aman Hussain, Liz Caperon, Alan M. Batt

**Affiliations:** 1 Department of Paramedicine, Monash University, Building H, Peninsula Campus, 47-49 Moorooduc Hwy, Frankston, VIC 3199, Australia; tyne.lunn@monash.edu (T.M.M.); brendan.shannon1@monash.edu (B.S.); cheryl.cameron@monash.edu (C.C.); 2 Department of Kinesiology, Gupta Faculty of Kinesiology of Applied Health, University of Winnipeg, 400 Spence Street, Winnipeg, MB R3B 2E9, Canada; am.hussain@uwinnipeg.ca; 3 Faculty of Environmental and Urban Change, York University, 4700 Keele St., North York, ON M3J 1P3, Canada; lizcaperon14@gmail.com; 4 School of Nursing, Queen’s University, 99 University Avenue, Kingston, ON K7L 3N6, Canada; 5 Institute for Health Policy, Management and Evaluation, University of Toronto, 155 College Street, Suite 425, Toronto, ON M5T 3M6, Canada

**Keywords:** community paramedicine, community needs, social needs, needs assessment, paramedic

## Abstract

**Background/Objectives:** Community paramedicine programs have existed since the early 2000s, and while resource optimization remains a predominant driver, innovation in recent years demonstrates that when community paramedicine is integrated into healthcare, it is well-positioned to support the needs of structurally marginalized communities by focusing services for those facing barriers to accessing equitable care. A recent scoping review described the evolving ways community paramedicine models are addressing health and social needs within communities around the world. We aimed to identify and explore existing community needs assessment tools in Canada to guide the initial development of a needs assessment tool for community paramedicine. **Methods:** We conducted a document analysis of existing community needs assessment resources to identify current tools or processes used to identify community needs, as well as determine gaps to address and support. Documents were collected for review via a targeted literature search of both published and gray sources, and direct document requests of community paramedicine service providers to review guides informing current service planning in Canada. We presented a draft of the tool to participants at a community paramedicine conference for their review and feedback, and we incorporated this feedback into the final version. **Results:** We reviewed 38 documents to identify and synthesize key elements within community health and social needs assessment tools and frameworks. Findings informed an interim Community Paramedicine Needs Assessment Tool (CPNAT) that the team presented to 112 community paramedicine experts and partners. We received 33 group responses of detailed feedback that we used to further refine and finalize the tool. **Conclusions:** The CPNAT can support enhanced health equity by guiding community paramedicine programs to better align services, policies, and funding with the health and social care needs of communities.

## 1. Introduction

**Figure 1:**
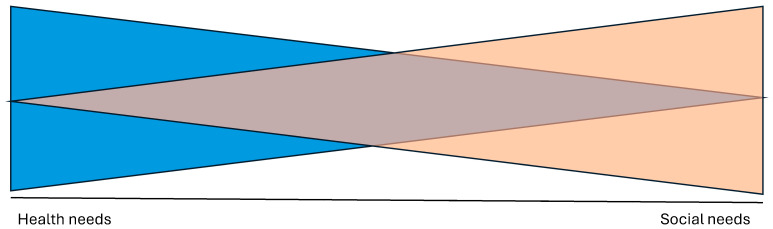
The health and social needs continuum.

Human health and social needs exist along a dynamic continuum. This continuum (Figure 1) illustrates that increases in health incidences may be related to unmet social needs, while those with social privileges and adequate support may experience less frequent or severe health incidences [1,2,3]. Recognizing that health status is inextricably impacted by social determinants of health, community paramedicine has both an opportunity and a responsibility to address social needs to reduce healthcare inequities [4,5].

Community paramedicine is a healthcare approach where paramedics expand their traditional emergency response roles to provide a broader spectrum of services within the community, such as providing and supporting care to community members with chronic diseases, structural barriers to accessing care, and socio-economic issues [6,7]. Instead of solely focusing on acute emergency care and transportation to hospitals, community paramedics utilize their medical expertise to deliver primary care, preventive services, and health education and promotion directly to individuals in their homes or community settings. Community paramedicine programs have existed since the early 2000s and have historically focused on resource optimization [8,9]. While this remains a predominant driver, innovation in recent years demonstrates that when community paramedicine is integrated into healthcare, it is well-positioned to meet the needs of structurally marginalized communities by focusing services on those facing barriers to accessing equitable care [10,11,12,13,14].

A recent scoping review by Lunn et al. explored how community paramedicine supports social needs along a health and social continuum [15]. The review described the evolving ways community paramedicine models are addressing health and social needs within urban, rural, and remote communities around the world. A key recommendation from the review was the need to meaningfully engage communities when developing programs to understand, co-design, and implement a model that addresses the specific needs of each community; in particular, rural communities may suffer from both a lack of community-based services and appropriately designed programs to address some of the community’s needs. However, there was a lack of evidence to guide this approach. The results highlighted the opportunity to determine best practices for conducting community needs assessments that include equitable partner engagement and harnessing community expertise.

Communities are experts in their health and social experiences and are, therefore, key partners in co-designing service delivery [16]. Where community paramedicine programs continue to develop programming without meaningful community engagement, they risk exacerbating health services inequities. Where health systems remain the sole architects, without understanding and addressing the needs of communities, there will be gaps in delivery and challenges in conducting meaningful evaluation. Conversely, co-designing community-led and integrated healthcare programming will enhance feasibility and sustainability, and ideally, promote equitable experiences and meaningful outcomes.

Applying a systems-thinking lens, we identified community paramedicine more broadly, as it is positioned within integrated healthcare systems, relating to how healthcare itself is positioned within greater society [17]. Recognizing that system complexities impact health status, paramedicine has a social responsibility to meaningfully engage in community needs assessments through co-designed service delivery and the generation of upstream health and social solutions for individuals and communities [5,10,17,18]. However, there remains a lack of guidance related to conducting needs assessments in community paramedicine, leading to inconsistencies in design, development, implementation, and evaluation of outcomes [15]. Due to the lack of appropriate needs assessment tools in paramedicine and the potential to learn from strategies implemented in other professions, this study aimed to identify elements of community needs assessments and use those elements to inform the initial development of bespoke guidance for those developing community paramedicine programs.

We conducted a multi-method study comprising a document analysis and feedback from system partners to achieve the following aims in the context of Canadian paramedicine:

1. Identify and explore existing community needs assessment tools informing community paramedicine and health/social services program development.
2. Identify gaps in community needs assessment strategies to enhance community-led and co-designed service delivery.
3. Produce draft materials to be presented at a meeting of community paramedicine system partners and gather their feedback to inform a final version.

## 2. Materials and Methods

### 2.1. Study Design

This manuscript describes a multi-phase project that aimed to produce the initial version of the Community Paramedicine Needs Assessment Tool (CPNAT). Since systems are vast and include geopolitical implications on health status, we set boundaries for this study to support feasibility. Therefore, this study focused on community paramedicine within Canada, in urban, rural, and remote contexts. However, the output from this study may have applicability in other contexts outside of Canada with careful considerations related to transferability (e.g., cultural context and societal expectations) and its inherent limitations.

### 2.2. Positionality

TM is an able-bodied, Canadian, white woman. She is a degree-educated paramedic, researcher, and a subject-matter and research expert on community paramedicine in Canada. BS is an able-bodied, Australian, white man who is a doctoral-educated paramedic, researcher, and educator with multi-methods methodological expertise and research expertise in community paramedicine. CC is an able-bodied, Canadian, white woman. She is a doctoral candidate, paramedic, researcher, and subject-matter expert on paramedicine in Canada. AH is an able-bodied, Canadian, East-Indian man who is a doctoral-educated researcher with methodological expertise in health professions education. LC is an able-bodied, British, white woman who is a doctoral-educated health researcher with methodological expertise and research expertise on community paramedicine in Canada, community health, and social determinants of health. AB is an able-bodied, Irish Canadian, white man who is a doctoral-educated paramedic, researcher, and educator with methodological expertise, including document analysis, and research expertise in community paramedicine. We acknowledge that our positionality as a team and as individuals means we approached this study from a specific lens. As such, it was important to engage with partners throughout the development of this tool to gain broader perspectives, diverse opinions, and rich feedback. While we approached this work from a pragmatic stance (with a specific aim to focus on an answer that can be implemented in the ‘real-world’ setting), we adopted a systems-thinking lens [17] that was sensitized by various critical social theories (whereby we acknowledge that healthcare services are shaped by historical, societal, and institutional forces that privilege some while marginalizing others) [19]. This includes insights from various theories that inform the social and structural determinants of health, such as structural theories of poverty, social cohesion, social exclusion, critical race theory, discrimination, and social dominance theory, and many others [20]. We do not seek to elevate any one of these theories over others, and we do not claim to have used any of these theories in their totality—rather, we are sensitized by the concepts of equity that each theory introduces to the work. These perspectives led us to intentionally focus on ensuring that equity-seeking measures were reflected throughout the development and subsequent output of the tool. We use the WHO Operational framework for monitoring social determinants of health equity as a means of categorizing elements within the tool, and its use is not intended to highlight specific determinants as more important than others.

### 2.3. Document Selection and Analysis

The review by Lunn et al. demonstrated a lack of peer-reviewed studies that align with the findings of community paramedicine programs internationally, and we, therefore, sought to also include gray literature [8,15]. A document analysis was considered the most appropriate choice aligned with the timelines, resources, and feasibility of this initial phase of project work to identify best practices. Document analysis is a qualitative research approach where texts are systematically explored and interpreted by the researcher to evoke meaning and understanding, and to generate knowledge relative to specific research question(s) [21,22]. While document analysis is often used in triangulation to complement other methodologies, it can be used as a stand-alone method [22]. We registered the project on the Open Science Framework (OSF) to enhance the transparency and trustworthiness of the study design and implementation. We report our findings using the Checklist for the Use and Reporting of Document Analysis (CARDA) [23].

Using O’Leary’s eight-step planning approach for document analysis [24], we identified the following documents for inclusion:

1. Community needs assessment in published and gray literature in community paramedicine and other health and social care professions in Canada, with a targeted literature review.
2. Community needs assessment tools used to guide program design and development by urban and rural community paramedicine service providers in Canada.

The results of these approaches were included and examined in the document analysis. We processed the texts using the eight-step approach outlined by O’Leary [24]. We asked questions about the texts and referred to this as the interview. The interview phase was conducted by TM. Questions were informed by the findings of Lunn et al.’s scoping review [15]. See Appendix A.1.

#### 2.3.1. Document Corpus

Documents were included as described above. Documents were also included if they were used to guide community paramedicine program design and development in Canada. Documents were excluded if they did not describe a community needs assessment, described an individual patient-level needs assessment, were duplicates, were not produced in English, or were unable to be collected for review.

#### 2.3.2. Document Collection and Management

We conducted a focused search of the literature to identify community needs assessment tools in health and social care professions in both published and gray literature. The search strategy involved searches with no date limitations in MEDLINE, CINAHL, and Google Scholar using combinations of the following phrases and keywords informed by the objectives of the study: needs assessment, community needs assessment, social needs assessment, conducting needs assessment, and community-level needs assessment. The gray literature search was guided by the Canadian Agency for Drugs and Technologies in Health (CADTH) gray matters checklist [25]. We conducted a backward citation chase of the literature search results [26]. We sought needs assessment tools and other program design documents via direct request from AB to members of the Canadian Standards Association (CSA) Group Technical Committee on Community Paramedicine Program Development via the CSA Communities website. This committee comprises experts in community paramedicine in Canada in urban, rural, and remote contexts. Full details on identifying and collecting the document corpus are outlined in Appendix A.2, including a PRISMA-flow diagram. Two reviewers screened each record, and disagreements were resolved via discussion. We identified a total of 26 full-text articles from our database search. These were combined with seven gray literature documents and two additional peer-reviewed studies supplied via the CSA Group Technical Committee on Community Paramedicine, two additional peer-reviewed studies identified during full-text review, and one additional gray literature source. This resulted in a total of 38 documents that were included for analysis. Documents were stored on a password-protected computer in a private folder. Documents were identified and collected by the team between January and March 2024.

#### 2.3.3. Document Quality

We found the documents to be of reasonable quality. Documents collected from database searches were comprehensive, in addition to those sourced from searching the gray literature. While some online tools comprised extensive chapters, other documents lacked structure. Documents were fully complete and free of obvious production gaps or redactions. We found that all documents provided content that directly addressed the research questions.

#### 2.3.4. Data Analysis

We performed abductive coding to identify key elements of community needs assessments. The data were analyzed for the prevalence of key elements, deductively coded against Lunn et al.’s scoping review findings, and inductively coded against the document data itself using Dedoose (v9.2.12). A codebook was created within Dedoose. Extraction and coding were performed by T.M., and a 20% sample was reviewed by A.M.B. Disagreements were resolved via discussion.

#### 2.3.5. Needs Assessment Domains

**Figure 2:**
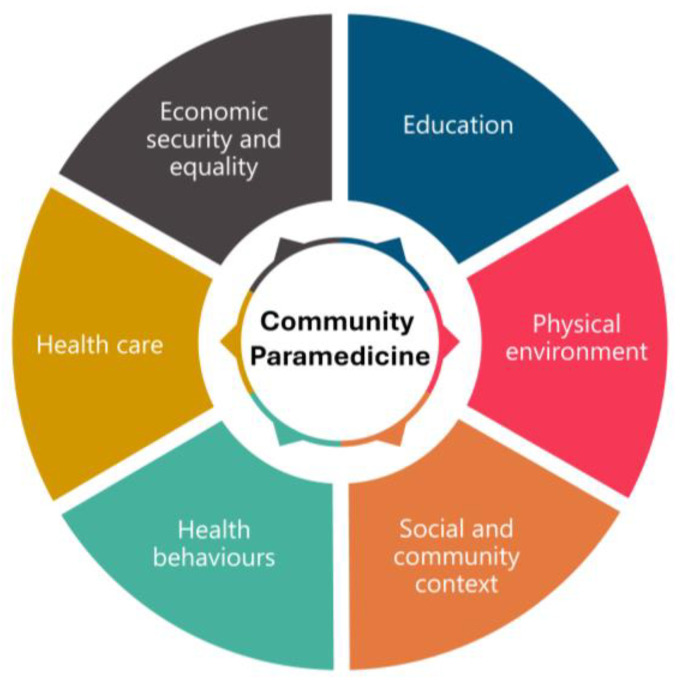
Community paramedicine domains mapped to the WHO social determinants of health equity domains.

We grouped elements according to the social determinants of health outlined in the scoping review and further refined these categories by grouping and collapsing common items to align with the World Health Organization (WHO) social determinants of health equity domains [27] (see Table 1 and Figure 2). This framework was intentionally selected to ensure that equal consideration was given to all determinants of health, and not just those traditionally engaged with by community paramedicine programs. The items were organized into their respective key concept areas by TM and reviewed by AB. We identified gaps by considering what was not present in the data (informed by a systems-thinking approach used and published as part of a larger pan-Canadian paramedicine project [17]) and identified elements in the scoping review by Lunn et al. [15] that did not appear in the data for this study. Interpretation of the data was completed in a summative process, while seeking to remain objective to the content and its relevance to the research questions.

The draft CPNAT was created by combining the findings of our existing scoping review and the extracted data from the document analysis—see Table 1 to review all key elements incorporated into the first draft of the CPNAT.

### 2.4. Feedback from System Partners

#### 2.4.1. Procedure

We presented the first draft of the CPNAT to system partners in community paramedicine at the 20th International Roundtable on Community Paramedicine (IRCP) meeting, which took place in June 2024 in Quebec City, Canada. While we did not gather individual demographic data from participants, the attendees at the 20th IRCP came from multiple countries (e.g., Canada, the United Kingdom, Australia, Republic of Ireland, and Germany), represented diverse voices across the sector (e.g., gender, ethnicity, Indigeneity, geographical spread, and education level), and were often in strategic positions related to community paramedicine (e.g., service leaders, program developers, academics, policymakers, and other healthcare professionals). The draft was distributed by email to conference attendees a week in advance of the feedback session. An explanatory statement on the nature of the session, along with a copy of the ethics approval letter, was also attached to this email. Participants were informed of the voluntary nature of their participation and their right to withdraw or not participate at any stage of the process. On the day, members of the project team facilitated a 60 min feedback session, with participants answering feedback questions. See Appendix A.2. During this 60 min period, participants worked in small groups to review and provide feedback on the draft CPNAT using an online Google form that was saved to a secure Monash Google Drive account. No recordings were made.

#### 2.4.2. Data Analysis

All data were collated and organized for review by the project team after the event. Responses from participants were reviewed individually and categorized via a high-level deductive content analysis [28] informed by the six CPNAT-related questions posed to the participants (i.e., structure of tool, concept clarity, missing elements, additional guidance, utility in context, and additional feedback). Where possible, responses were matched directly to existing codes developed in the document analysis to guide possible edits. All feedback from the system partners was initially reviewed by TM and AB, and tracked edits were made to the draft CPNAT to reflect the feedback. Feedback was then shared with all team members, along with a second draft of the CPNAT to review and provide additional input prior to finalizing the tool. This feedback process resulted in minor edits to the structure and content of the tool, in addition to a plan for resources to support its use and plans for future developments, such as a web app. Examples of feedback and how they influenced the final tool are provided in Appendix A.3.

### 2.5. Finalized Tool

The final draft of the CPNAT was submitted to Healthcare Excellence Canada (HEC), where it underwent editorial review by their Northern and Indigenous Health Team. Several edits were made to clarify the language used and the intent of certain questions. Upon final approval of the CPNAT by the research team, HEC facilitated the typesetting, design, and translation of the document into French for publication. The working published version of the CPNAT can be accessed for free in English (https://tinyurl.com/en-cpnat (accessed on 12 March 2025)) and French (https://tinyurl.com/fr-cpnat (accessed on 12 March 2025)) on the HEC website (Note: We made minor editorial changes to participants’ quotes in this manuscript (e.g., correction of spelling errors, capitalization) but did not change the content, structure, or words).

### 2.6. Trustworthiness and Validity

To promote the trustworthiness [29] of our study findings, we explored the documents’ agendas and biases, such as who produced them and the audience the documents were intended for, based on criteria such as relevance and reliability as described by O’Leary [24]. We conducted our research using a systematic approach for planning and conducting document analyses described by O’Leary, and reported our findings using the CARDA checklist, all underpinned by a reflexive approach [30]. Data were organized and stored in password-protected archives. We spent time engaging with and revisiting the data over multiple months to support credibility. Dependability was maintained through abductive coding, including a rationale for code creation in alignment with pre-existing frameworks. Reproduction and confirmability were ensured by publicly registering our project on OSF (https://osf.io/2d9j6/ (accessed on 12 October 2025)) and supporting alignment through a clear audit trail of detailed notes related to data sources, comments, and changes from the first draft of the CPNAT through to the published version.

The face validity of the tool and initial content validity are supported based on our multi-phase development process and the feedback received from the IRCP attendees (in particular, regarding Questions 1, 2, and 3 in Appendix A.3). However, we acknowledge that we did not undertake any structured validation phase as part of this project. The CPNAT is currently in use in over 40 test sites, and results from these use cases will be available in 2027. These results will inform revisions to ensure the CPNAT is a reliable and implementable tool.

### 2.7. Ethics Approval

Ethics approval was provided by the Human Research Ethics Committee at Monash University (#42775).

## 3. Results and Findings

### 3.1. Document Analysis

Of the 38 included documents, 25 were published in the United States of America, 9 in Canada, and 1 each in Italy, Israel, Jamaica, and South Korea (based on a project in Vietnam). Documents identified for inclusion were published between 2001 and 2023. They comprised a mix of reports, guidelines, reviews, and tools, and all were available in a digital format. Ten documents were rural in focus, with the remainder focused on regional, urban, or identity-based communities. See Supplementary Material S1 for detailed extraction data and document characteristics.

Six of the nine documents published in Canada (67%) explored community needs assessment in the context of Indigenous reconciliation and health justice [31,32,33,34,35,36]. These key elements identified in the provision of reconciliation informed a designated section guiding considerations for equity and Indigenous health justice within the draft CPNAT. While intersectionality was identified in the scoping review as an important area to explore within paramedicine, with implications that impact health status, outcome, and healthcare experiences, considerations of intersectionality in needs assessment approaches remained a gap throughout this review, with only seven documents (18%) outlining considerations of intersectionality in needs assessment approaches [32,33,37,38,39,40,41].

### 3.2. System Partner Feedback

A total of 33 group responses were received from 112 participants at the feedback session. Twenty-eight groups provided detailed feedback on the structure and content of the tool. For example, participants spoke of the ability of the tool to provide an overview of the health and social care system, stating, “*it is very comprehensive. It covers all key areas that allow you to understand all aspects in health and non-health partnerships, service delivery, and system gaps.*” In relation to its ability to provide the information that they, as developers of CP programs, would need, participants were resounding in their support for the tool, stating it “*appears to provide a robust assessment of community overview and needs from various lenses and perspectives.*”

However, using the tool would present challenges to some. For example, some highlighted the need for “*an option to skip sections that aren’t needed*”, while others stated that the tool’s “*length makes building a case in your head dynamically hard to retain*”. In its attempts to be comprehensive, some felt that the tool “*is trying to determine too many things. It seems like it didn’t match a need assessment with program delivery.*” Despite its comprehensiveness, some participants highlighted areas where the tool may struggle to adequately or accurately capture diverse community needs, such as transient populations, increasing vaccine hesitancy, and poor health literacy, among others.

A total of 21 group responses suggested the need for additional guidance and resources to use the tool, such as a user toolkit. Several groups suggested the need for a completed example community needs assessment, or, at a minimum, examples of the types of answers that the tool might generate. Several participants raised questions related to scalability; for example, “*How do we apply the tool to specific communities? Specific buildings that we serve?*”. Participants provided feedback that guidance on what to do with the completed tool and the answers within was required, e.g., how does the information lead to supporting a gap analysis, what is the process for ranking answers, and how should they use this information to guide program development.

Finally, there were several suggestions to develop a branching logic software-based version of the tool to encourage uptake and increase applicability in the future. These would help to categorize the importance of certain answers to specific communities or populations via a “*smart form that skips repetitive or irrelevant sections and brings you to the next most important piece.*” The potential to integrate existing data sources (both publicly available and privately held) and machine learning into a future digital tool was also mentioned by some groups. Several attendees signaled their intent to use the CPNAT in their system once developed and their willingness to provide feedback to inform future developments.

## 4. Discussion

This study aimed to identify and explore community needs assessment tools informing community paramedicine and health/social service program development. Through a document analysis and the input of system partners, we drafted a needs assessment tool for use by community paramedicine programs. Informed by our analysis, we discerned the need for community paramedicine programs to plan and co-design needs assessments with communities, the need to intentionally assess for inequities while creating these assessments, and opportunities for future directions.

Community paramedicine programs are not off-the-shelf solutions to addressing unmet health and social needs [42]. Upstream solutions should be tailored to align with community needs. Recent reviews of community paramedicine program development indicated a lack of structured tools to facilitate such assessments and an ongoing need for such tools to prevent blind spots when developing programs [15]. If we continue to remain unaware of community needs—from the community’s perspective—while we innovate and expand community paramedicine programs, we may not be optimally designing such programs to meet community needs. At best, this risks inappropriate and inefficient resource use, and at worst, we may be directly exacerbating health inequities. Therefore, it is imperative that communities are lead partners in the co-design of needs-based services from the outset of community paramedicine program development. However, meaningful community engagement takes time, can face significant barriers, and requires collaboration [43,44,45].

Planning allows for the involvement of key partners from the beginning of the process, cultivating trusting relationships and community support that is essential for empowering community agency and leadership [46]. Community involvement can enhance outcomes by generating community-based participatory research evidence [47,48]. Through planning, key partners in allied health and social services can be identified to contribute and share in the effort. These relationships will be valuable for collaborating to generate strategies for services to address community needs and reduce inequities. The comprehensive outcomes of this project also highlight the need to prioritize detailed planning when conducting a community needs assessment. Developing a plan for assessing community needs will support service leaders in understanding how to best serve their communities in logical, efficient, and prioritized ways [47,49]. This requires clearly identifying the purpose of the needs assessment. Despite the need for continued insights to inform the evolution of the CPNAT, the application of the tool in its current conceptual state can provide trustworthy outcomes—but it will require significant support in the form of meaningful engagement strategies and appropriate funding [50]. Robust assessment will take time—it will need to be thorough, iterative, flexible, and responsive to community partner capacity and priorities. Paramedic service leaders should be flexible with timelines to support community agendas.

Another challenge for those who use the CPNAT is how to prioritize identified needs. Outcomes of the CPNAT are non-prescriptive, and applying the tool may yield valuable findings to inform strategies that better align services with community needs. It is imperative to identify community priorities to establish trust and respect, especially when these are misaligned with health system priorities. We also offer that there is an opportunity here for service leaders to identify strategies that support collaboration and integrate care with other health- and social-care professions—not all community needs are best met by community paramedicine programs [15]. Identifying allied health, social, and community partners, and populations with needs and their caregivers, in the planning stage and mitigating barriers to participation, will enable community-led and co-designed outcomes and priorities [48]. Applying a strengths-based approach to identify and support existing community initiatives that focus on outcomes of health justice for structurally marginalized groups will help to ensure that any identified priorities are meaningfully addressed by community paramedicine programs in collaboration with other health and social care services [51,52].

We must, however, acknowledge that the evolution of community paramedicine has been largely driven by health system utilization measures, policies that do not engage meaningfully with communities, and health system structures that vary greatly and are deeply embedded [9]. Recognizing that healthcare in Canada is ultimately inherited and structured by colonialism, health-system-driven initiatives risk overlooking inequities in healthcare experiences and health outcomes [53,54]. Therefore, it is imperative that the CPNAT evolves to become a core tenet in the development of programs moving forward, with the intention of considering intersectionality when assessing for inequities to reduce barriers and promote health justice for structurally marginalized populations [15,55,56]. For example, there is an identified need within Canada for ongoing commitments of reconciliation that support Indigenous communities’ self-determination and that they be restored with agency and autonomy. One way to enact this commitment is by engaging Indigenous communities in co-design, or better yet, to co-create community paramedicine programs serving their communities [57]. To not do so means that health and social program development remains largely health-system-driven and designed, risking further embedding settler colonialism and structures that promote inequity.

### 4.1. Example Use Case

The CPNAT was used by one community in Ontario, Canada, where the developers implemented the CPNAT via a strategic committee of community partners providing social services. This committee worked through the sections of the CPNAT in an intentional manner, recruiting others from the community where needed to provide appropriate insight into the community’s capacity, resources, and perceived needs. Data collection involved census data, paramedic service call data, data from national health registries for the region, and insights from paramedics. Community members were engaged through town hall meetings and via community events. The findings of the CPNAT informed the initial need for priority social prescription pathways related to food insecurity and social isolation. These are now being created with community service partners.

### 4.2. Limitations

The document analysis was limited to those documents that could be collected. While we sought to be comprehensive in identification and collection methods, there may be additional documents that exist but are not available in the public domain. We limited the review to English-language documents. We acknowledge that the inclusion of English-only documents risks excluding potentially useful information from authors or jurisdictions where English is not the primary language. We acknowledge that we did not use community-based participatory approaches when developing the CPNAT and relied on consultative approaches due mainly to project constraints (e.g., academic timelines, funding period). We did, however, seek to engage with system partners and offer the CPNAT in its current form as a conceptual foundation on which future work can build, enhancing the tool. We acknowledge our own inability to ensure equity, representation, and shared decision-making based on our lack of demographic insight into the system partners at IRCP. Future improvements to the tool should use equity-serving, co-design, and participatory research approaches that involve communities. As such, we published the CPNAT in its current form under a Creative Commons license to facilitate future enhancements and iterations by the community. We also acknowledge that we did not undertake any structured validation phase as part of this project. We offer that feedback from current users of the tool will provide the required validity evidence to move this tool from a conceptually useful model to one with real-world evidence to support its use. Any formal updates to the tool will be hosted on the HEC website with versioning control and insights from such validation exercises. Other adaptations in other contexts will require similar efforts by those in those jurisdictions.

### 4.3. Future Directions

Despite direct outreach to community paramedicine services in Canada to explore current tools used in program design and development, less than 25% of the documents were collected from this source. This may be reflective of the reality that service leaders in Canada are limited by operational demands, budget cycles, policy momentum, or disruption, and are often required to develop programming strategy in the absence of standards or guidance [9]. This does not discredit initiatives created in this order; rather, it recognizes the need to apply evidence-informed guidance to ensure the quality of initiatives once available. Community paramedicine continues to be well-positioned within healthcare to be responsive to the dynamic evolution of community needs. Community paramedicine leaders are now supported with this tool to inform community needs- and values-based care models. The CPNAT provides a structured tool to guide community needs assessment and will enhance health equity by supporting alignment of community needs and services. However, note that this does not replace the need to measure indicators of health equity in the community, such as those outlined in the WHO Operational framework for monitoring social determinants of health equity.

The next iterative steps of this multi-phase project include opportunities to improve the application and accessibility of the tool through formatting, translation, testing, validation, evaluation, and ongoing improvement. Equity-focused implementation strategies and community-based participatory research methods should be applied going forward in the continued development and validation of the CPNAT, including data stewardship [48,52,58]. This equity-focused lens should continue when synthesizing tool outcomes into service initiatives. Ideally, the decision-making processes applied to optimize the CPNAT and produce equity-generating initiatives will be participatory and inclusive [48,59]. An opportunity exists for researchers and service leaders to explore the transferability and adaptability of the CPNAT for use in alternate jurisdictions, beyond Canada. Such transferability must be performed intentionally and carefully, as cultural differences, differences in service availability, and societal expectations of community paramedicine may differ significantly.

## 5. Conclusions

This study involved a document analysis that identified and synthesized community health and social needs assessment tools and frameworks. Feedback from system partners on these synthesized findings informed the initial development of a needs assessment tool for community paramedicine. The development of this conceptual tool was prioritized in response to an identified need for a structured guide to approach community needs assessment to inform community paramedicine program design and delivery. The continued use and validation of the CPNAT will influence improved service design, inform commissioning choices, and support resource allocation for marginalized communities to improve health equity by ultimately guiding community paramedicine programs to better align their services with the health and social care needs of communities.

## Figures and Tables

**Table 1:** Key elements of community health and social needs assessment, informed by the WHO Operational framework for monitoring social determinants of health equity.

Social Determinant of Health Equity (SDHE) Domains	Operational Definition	Key Elements of Community Needs
Economic security and equality	Ensures access to sufficient financial resources, reducing income inequality to improve health outcomes.	Employment Food security Income Poverty
Education	Promotes equitable access to quality education, as higher levels of education are linked to better health outcomes.	Education access Education quality Education outcomes
Physical environment	Focuses on providing healthy living conditions, including clean air, safe housing, and access to green spaces.	Air quality and climate Disasters Energy, fuels, and technologies Housing Land tenure Road safety Water, sanitation, and hygiene Urbanization
Social and community context	Emphasizes the importance of strong social networks, community engagement, and social support systems in promoting health equity.	Conflict, crime, and violence Discrimination Forced displacement, migration Gender equality and women’s empowerment Healthy aging Incarceration Social support
Health behaviors	Targets the adoption of healthier lifestyles, including nutrition, exercise, and reducing harmful behaviors like smoking and alcohol misuse.	Substance use Physical activity Nutrition
Healthcare (health services)	Ensures access to quality, affordable healthcare services that meet the needs of all population groups, regardless of socioeconomic status.	Healthcare access and affordability Health system (healthcare)

**Figure A1:**
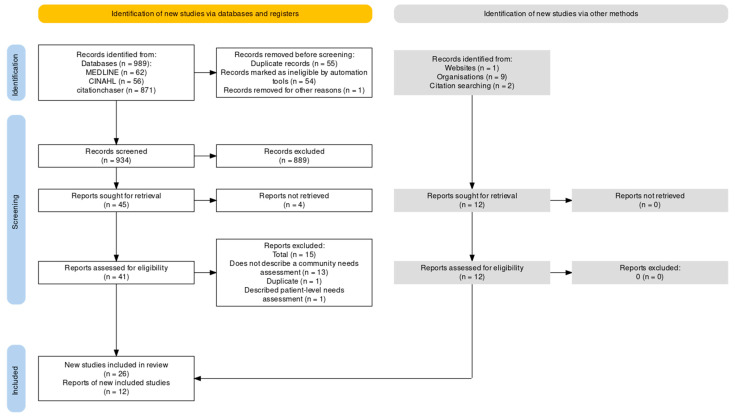
PRISMA Flow Diagram.

## Data Availability

All data related to this study is archived on the OSF project page at https://osf.io/2d9j6/ (accessed on 12 October 2025).

## Supplementary Materials

The following supporting information can be downloaded at https://www.mdpi.com/article/10.3390/nursrep15120440/s1, File S1. Extracted Data.

## Public Involvement Statement

No public involvement in any aspect of this research.

## Guidelines and Standards Statement

This manuscript was drafted against the CARDA Guideline for document analysis research [23].

## Abbreviations

The following abbreviations are used in this manuscript:

CPNAT	Community Paramedicine Needs Assessment Tool
CARDA	Checklist for the Use and Reporting of Document Analysis
CINAHL	Cumulative Index of Nursing and Allied Health Literature
CADTH	Canadian Agency for Drugs and Technologies in Health
CSA	Canadian Standards Association
WHO	World Health Organization
HEC	Healthcare Excellence Canada
OSF	Open Science Framework
CP	Community Paramedicine
IRCP	International Roundtable on Community Paramedicine

## Appendix A

### Appendix A.1. Interview Questions for Document Analysis

Determining the questions to ask of the texts was informed by the scoping review of Lunn et al. We applied the interview technique to ask the following questions:

1. Who produced the text?
2. When/where was the text produced?
3. What is the collection source of the text?
4. What type of document is the text?
5. Who is the intended audience?
6. Who is the community served (social unit)?
7. What is the community description?
8. Who are the community partners (stakeholders)?
9. Are there any Indigenous community considerations?
10. What does the text say and what is the context?
11. What are the elements included in the needs assessment related to health and/or social needs?
12. Is intersectionality considered in the text?
13. Are there any obvious gaps in health and/or social care needs assessment items?

### Appendix A.2. Search Strategy and Results

The search strategy from Lunn et al. [15] was used, combined with specific searches using combinations of the following terms: “Needs assessment; Community needs assessment; Community health needs assessment; health needs assessment; community-level needs assessment; Strengths-based approach; Partner(s); Partnership(s); Stakeholders; Co-design” in MEDLINE, CINAHL, and on various organizational websites as guided by CADTH, Google Scholar, and Google web searches. Searches were performed in February 2024.

### Appendix A.3. Feedback Questions

Please review the CPNAT in your groups. In addition to reviewing and providing feedback on individual elements of the CPNAT, please provide your answers to the following questions:

1. Does the structure of the tool make sense?
2. Are the concepts explained clearly in the tool? If not, please provide suggestions for improvements.
3. Is there anything missing from the tool that should be included? Please provide details.
4. What additional guidance would you need to be able to use this tool?
5. Do you see the tool being useful in your context? Please provide details.
6. Is there any additional feedback you would like to give on the tool?
7. What is needed to support the uptake and implementation of the Community Paramedic Needs Assessment Tool?
  ○ Do you see a role for HEC in this?
8. Is there interest in partnering with HEC to help develop and support pathways to spread community paramedics innovations?

### Appendix A.4. Examples of Feedback and Changes to CPNAT

**Table A1:** Examples of Feedback and changes to CPNAT.

Source of Feedback	Original Content in First Draft	Feedback Received	Changed Content in Final Draft
IRCP attendee	Short Questions to ask prior to using the CPNAT section.	Further support ‘Questions to ask prior’ and ‘Preparation’.	Expanded and included additional planning sections related to Getting Started, Community Partners, and Cultural Safety
IRCP attendee	Equity and reconciliation questions focused mainly on Indigenous people.	Expanding the section on equity and reconciliation beyond Indigenous people and making it more general to address more at-risk or historically marginalized populations.	Specific references made to other marginalized and structurally vulnerable groups in the tool, including considerations related to immigration status, sexual orientation, gender identity,
HEC Northern and Indigenous Health team	Indigenous governance (e.g., Chief and Band Council).	Remove Chief and Band Council and just have “First Nations, Inuit or Métis governance” because Inuit and Métis governance looks different and will not be easily described in a few words.	First Nations, Inuit, or Métis governance.
IRCP attendee	Not linked to funding opportunities.	Could the tool link to funding opportunities available in our area.	Section added to Follow Up to align with Future Initiatives and Budgets.
