# Supplementary material for: Developing the Community Paramedicine Needs Assessment Tool
Source: Nurs Rep. 2025 Dec 10;15(12):440. doi: 10.3390/nursrep15120440 (PMC12735474; doi:10.3390/nursrep15120440)
Supplement: Supplementary file 1 [file nursrep-15-00440-s001.zip › S1. Extracted Data.pdf]

Supplementary Information S1—Extracted Data

Author / Year	Country	Language	Document Title	Document Type(s)	Intended Audience(s)	Community Served (social unit)	Community Description	I. Community and stakeholders	II. Indigenous community considerations	III. Existing information	IV. Access to health and social care services	V. Daily living considerations	VI. Mental health and substance use	VII. Technology	VIII. Social support agency and belonging	Is intersectionality considered in the document?	Additional relevant content or context to include and consider in the development of a community needs assessment?
2014 Sandra Kurdziel	Canada	English	Social Navigator Program - An Overview	Report.	Healthcare, Social Services, Community Services, and Public Safety Services (e.g., police, fire).	Structurally Marginalized (e.g., disabled, elderly, unhoused, refugee, migrant, immigrant, incarcerated, etc.) Geographical – Urban.	Hamilton's downtown area; people who use drugs; mental illness; poverty; homelessness; criminal activity/repeat police interaction; social exclusion; general low SES.	3. Outline stakeholders who should be involved with and/or consulted when conducting the community needs assessment.		Are specific vulnerabilities or characteristics associated with this population that may result in marginalization (e.g., minors, children in care, incarcerated, victims of human trafficking, sex workers, undocumented or illegal immigrants)? Is this community located in an urban, suburban, rural, mixed, isolated geographic setting, or bedroom community? Existing information to gather on the community (data, as applicable).	What healthcare access points exist in the community, what services do they provide, and are they accessible and equitable? What social care access points exist in the community, what services do they provide, and are they accessible and equitable? What social services exist in the community, what services do they provide, and are they accessible and equitable? Do people experience homelessness in the community?	What is the status of SDH for the community? Income, Housing, and Food Security.	Are specific mental health concerns present within the community?				
2020 County of Renfrew Paramedic Service	Canada	English	Community Needs Assessment - Wellness Clinics	Guideline.	Healthcare.	Structurally Marginalized (e.g., disabled, elderly, unhoused, refugee, migrant, immigrant, incarcerated, etc.) Geographical–Regional Priority Populations: orphaned patients; chronic medical conditions; mental health; frailty; high risk of falls; diabetes; substance use.	County of Renfrew, Ontario; predominantly rural; includes lakes, rivers, natural spaces, and farmland; significant forestry industry; aging population >65; chronic illness; mental health; people who use drugs; limited access to primary care.	2. Are there specific racial or identity considerations for the community? 3. Outline stakeholders who should be involved with and/or consulted when conducting the community needs assessment: primary care; geriatric care; OT/PT; diabetes educators; community mental health; and family health teams.		Is this community located in an urban, suburban, rural, mixed, isolated geographic setting, or bedroom community? Existing information to gather on the community (data, as applicable).	What healthcare access points exist in the community, what services do they provide, and are they accessible and equitable?	What is the status of SDH for the community? Income, Housing, Food Security, Education, Employment, Public Transport, Infrastructure, and Early Childhood Development; Age (older adults, >65); gender identity/expression; sexual orientation/attraction.	Are specific mental health concerns present within the community? Is there a mental health facility accessible to the community (including inpatient services)?		Are social activities accessible within the community? Are social activities specifically aimed at adults over age 65? Are there specific cultural or ethnic considerations for the community?	Yes	Objective of this document is to be used to guide a Community Needs Assessment to inform decision-making on determining ideal locations to establish Wellness Clinics in the county, prioritizing geographic vulnerability and priority populations.

Supplementary Information S1—Extracted Data

Author / Year	Country	Language	Document Title	Document Type(s)	Intended Audience(s)	Community Served (social unit)	Community Description	I. Community and stakeholders	II. Indigenous community considerations	III. Existing information	IV. Access to health and social care services	V. Daily living considerations	VI. Mental health and substance use	VII. Technology	VIII. Social support agency and belonging	Is intersectionality considered in the document?	Additional relevant content or context to include and consider in the development of a community needs assessment?
BCEHS	Canada	English	CP Community Selection	Guideline.	Healthcare (includes paramedicine services).	Structurally Marginalized (e.g., disabled, elderly, unhoused, refugee, migrant, immigrant, incarcerated, etc.). Identity-based (e.g., race, ethnicity, gender, sexual orientation, religion, etc.). Geographical - Rural, Geographical - Remote, rural, small rural, and remote.	Community defined as rural, small rural, and remote as per the MoH Rural Health Services in BC: A Policy Framework to Provide a System of Quality Care, 2015.	2. Are there specific racial or identity considerations for the community?	General assessment for impact on surrounding Indigenous communities.	Is this community located in an urban, suburban, rural, mixed, isolated geographic setting, or bedroom community? Existing information to gather on the community (data, as applicable).		Age, older adults/>65.				No	
Weeneebayko Area Health Authority (WAHA)	Canada	English	WAHA Paramedic Service's Indigenous Community Paramedic Program: Assessment of Health and Social Needs	Guideline.	Healthcare (includes paramedicine services).	Structurally Marginalized (e.g., disabled, elderly, unhoused, refugee, migrant, immigrant, incarcerated, etc.). Identity-based (e.g., race, ethnicity, gender, sexual orientation, religion, etc.).	Indigenous patients in James Bay communities, including Moosonee, Moose Factory, Fort Albany, Kashechewan, Attawapiskat, and Peawanuck, Ontario.	2. Are there specific racial or identity considerations for the community? 3. Outline stakeholders who should be involved with and/or consulted when conducting the community needs assessment.	Do established reciprocal relationships exist between health and social care professionals and Indigenous Peoples and their communities? What cultural considerations must be incorporated into health and social care provision? Are traditional ceremonial processes respected and conducted? Are Traditional Knowledge Keepers engaged in the design and provision of health and social care services? Are there Indigenous facilities, centers, or lodges? What languages are spoken among the community? Is time allowed in health and social care provision to tell and listen to stories? Is information related to health and social care services provided in a format and	Are specific vulnerabilities or characteristics associated with this population that may result in marginalization (e.g., older adults/>65, minors, children in care, incarcerated, victims of human trafficking, sex workers, undocumented or illegal immigrants)? Do language or communication considerations exist within the community? Existing information to gather on the community (data, as applicable).		What is the status of SDH for the community?				No	The objective of WAHAPS Indigenous Community Paramedic Program is to design a culturally sensitive and community-centered approach to assess the health and social needs of Indigenous patients in our James Bay communities, including Moosonee, Moose Factory, Fort Albany, Kashechewan, Attawapiskat, and Peawanuck. This program aims to improve healthcare outcomes by understanding and addressing the unique challenges faced by Indigenous communities.

Supplementary Information S1—Extracted Data

Author / Year	Country	Language	Document Title	Document Type(s)	Intended Audience(s)	Community Served (social unit)	Community Description	I. Community and stakeholders	II. Indigenous community considerations	III. Existing information	IV. Access to health and social care services	V. Daily living considerations	VI. Mental health and substance use	VII. Technology	VIII. Social support agency and belonging	Is intersectionality considered in the document?	Additional relevant content or context to include and consider in the development of a community needs assessment?
									manner that is appropriate to the community? Implement a comprehensive cultural competency training program for community paramedics. Educate paramedics on the historical context, traditions, and beliefs of the local Indigenous community. Foster an understanding of the social determinants of health in Indigenous populations.								
2014 Maureen Evashkevich & Michael Fitzgerald	Canada	English	A Framework for Implementing Community Paramedic Programs in British Columbia	Framework.	Healthcare (includes paramedicine services), and Government.	Geographical - Rural, Geographical - Remote, Geographical - Regional, and Geographical – Provincial.				Are specific vulnerabilities or characteristics associated with this population that may result in marginalization (e.g., older adults/>65, minors, children in care, incarcerated, victims of human trafficking, sex workers, undocumented or illegal immigrants)? Is this community located in an urban, suburban, rural, mixed, isolated geographic setting, or bedroom community? Existing information to gather on the community (data, as applicable), workforce data; and jurisdictional organization.		What is the status of SDH for the community?				No	
2017 CSA Group	Canada	English	Community paramedicine: Framework for program development (Z1630-17)	Framework	Healthcare (includes paramedicine services), and Government	All of the above.	Any community where a community paramedicine program is being	3. Outline stakeholders who should be involved with and/or consulted when		Is this community located in an urban, suburban, rural, mixed, isolated	What healthcare access points exist in the community, what services					No	

Supplementary Information S1—Extracted Data

Author / Year	Country	Language	Document Title	Document Type(s)	Intended Audience(s)	Community Served (social unit)	Community Description	I. Community and stakeholders	II. Indigenous community considerations	III. Existing information	IV. Access to health and social care services	V. Daily living considerations	VI. Mental health and substance use	VII. Technology	VIII. Social support agency and belonging	Is intersectionality considered in the document?	Additional relevant content or context to include and consider in the development of a community needs assessment?
							developed in Canada.	conducting the community needs assessment: Housing agencies/organizations; volunteer organizations.		geographic setting, or bedroom community? Existing information to gather on the community (data, as applicable).	do they provide, and are they accessible and equitable?						
2021 Ontario Ministry of Long-Term Care	Canada	English	Community Paramedicine for Long-Term Care: Framework for Planning, Implementation, and Evaluation	Framework.	Healthcare (includes paramedicine services), Government.	Structurally Marginalized (e.g., disabled, elderly, unhoused, refugee, migrant, immigrant, incarcerated, etc.) and Geographical – Provincial.	Ontario: individuals who are waiting for placement in a long-term care (LTC) home or who are soon to be eligible for long-term care.	Outline stakeholders who should be involved with and/or consulted when conducting the community needs assessment.	Do established reciprocal relationships exist between health and social care professionals and Indigenous Peoples and their communities?	Existing information to gather on the community (data, as applicable).						No	
2023 John Taplin	Canada	English	Exploring paramedic care for the First Nations in Alberta: a qualitative study	Study (peer-reviewed).	Healthcare (includes paramedicine services), and Research/Academia.		Indigenous members of First Nations in Alberta.	Are there specific racial or identity considerations for the community?	Study outlined 3 major themes specific to Indigenous experiences: racism, systemic barriers, and solutions.							Yes	Experiences of racial discrimination; lack of alternate care options led to accessing paramedicine services; lack of ability to return home if needing to access services outside of the FN; burden of additional work to act as cultural mentors to non-Indigenous providers; moral distress of paramedics when observing discrimination that interfered with care.
2020 Ruggles	United States	English	Frameworks for Community Impact - Community Case Study	Study (peer-reviewed).	Healthcare (includes paramedicine services), Community Services, and Research/Academia.	Geographical - Rural, Geographical - Regional/County.	NVRH is located in Vermont's Northeast Kingdom, a region known for its rugged rural landscape and independent and spirited people. The primary service area for NVRH is Caledonia and southern Essex counties, with just under									No	Introduced the Northeastern Vermont Regional Hospital (NVRH) Community Health Needs Assessment.

Supplementary Information S1—Extracted Data

Author / Year	Country	Language	Document Title	Document Type(s)	Intended Audience(s)	Community Served (social unit)	Community Description	I. Community and stakeholders	II. Indigenous community considerations	III. Existing information	IV. Access to health and social care services	V. Daily living considerations	VI. Mental health and substance use	VII. Technology	VIII. Social support agency and belonging	Is intersectionality considered in the document?	Additional relevant content or context to include and consider in the development of a community needs assessment?
							30,000 people. Population density in Caledonia County is 48.1 persons per square mile, and 9.5 persons per square mile in Essex County. Both counties are bordered by the Connecticut River and New Hampshire to the east.										
2021 Northeastern Vermont Regional Hospital	United States	English	Community Health Needs Assessment	Report.	Healthcare (includes paramedicine services), Social Services, Community Services, Government.	Structurally Marginalized (e.g., disabled, elderly, unhoused, refugee, migrant, immigrant, incarcerated, etc.). Identity-based (e.g., race, ethnicity, gender, sexual orientation, religion, etc.). Geographical - Rural, and Geographical - Regional/County.	Vermont's Northeast Kingdom; rural region; just under 30 000 people; 25-bed hospital; rugged physical environment.	Are there specific racial or identity considerations for the community? Outline stakeholders who should be involved with and/or consulted when conducting the community needs assessment, community experts with special knowledge; "community assets" categories (Our Partners and Resources): healthcare and complimentary health; human services; MH and substance use; older adults; economic development; schools; food cycle; faith-based; housing; arts and humanities; physical activity; recreational waterways; other local or regional resources/attractions; and media.		Are specific vulnerabilities or characteristics associated with this population that may result in marginalization (e.g., older adults/>65, minors, children in care, incarcerated, victims of human trafficking, sex workers, undocumented or illegal immigrants)? Is this community located in an urban, suburban, rural, mixed, isolated geographic setting, or bedroom community? Do language or communication considerations exist within the community? Existing information to gather on the community (data, as applicable), Suicide data; primary care to population ratio; mental health data; and primary care visits.		Income, Housing, Food Security, Employment, Public Transport, Air Quality, Drinking Water, Access to Communications (see Section VII).	Are specific mental health concerns present within the community? Is there a mental health facility accessible to the community (including inpatient services)?	What platforms or solutions are the community currently using? Are any community members disproportionately affected by a lack of access to technology? What barriers does the community face in using digital technology or tools?	Are social activities accessible within the community?	Yes	Key Health Equity Indicators - median income; people in poverty; income inequality; language spoken in the home; sexual orientation/gender identity; people with disabilities; access to healthcare; high school graduation rate; food environment index; severe housing problems; households with a computer; households with broadband internet; environmental factors (air pollution, drinking water)
2022 Franz et al	United States	English	Overview of the Most Commonly Identified Public Health Needs and Strategies in a Nationally Representative	Study (peer-reviewed)	Healthcare (includes paramedicine services), Community Services, Government, Research/Academia		Nationally representative sample of nonprofit hospital Community Health Needs Assessments										Table 1. Definitions and Categorization of Community Health Needs, categories: mental health; substance use; SDOH;

Supplementary Information S1—Extracted Data

Author / Year	Country	Language	Document Title	Document Type(s)	Intended Audience(s)	Community Served (social unit)	Community Description	I. Community and stakeholders	II. Indigenous community considerations	III. Existing information	IV. Access to health and social care services	V. Daily living considerations	VI. Mental health and substance use	VII. Technology	VIII. Social support agency and belonging	Is intersectionality considered in the document?	Additional relevant content or context to include and consider in the development of a community needs assessment?
			Sample of Nonprofit Hospitals														preventative health; access to care; obesity; chronic disease; cancer; senior health; maternal and child health; heart disease; tobacco; asthma; pediatrics; infectious disease.
2021 Loo et al	United States	English	Understanding community member and healthcare professional perspectives on gender-affirming care—A qualitative study	Study (peer-reviewed).	Healthcare (includes paramedicine services), and Research/Academia.		transgender and gender diverse (TGD) people in rural counties in Massachusetts, New York, Connecticut, Vermont, and New Hampshire, USA.					gender, transgender, and gender diverse.				No	Results: Both community members and HCPs spoke of the need for connectedness and linkages among disparate health system components for gender-affirming healthcare. Participants expressed this priority through calls for systems-level improvements within existing services (e.g., expanded data collection, expanded mental health services, inclusive and affirming health care environments, and TGD staff). They also expressed the need for expanded TGD community outreach and engagement (e.g., incorporation of a patient feedback process, TGD health navigators, and resource mapping).
2018 Velonis et al	Canada	English	“One program that could improve health in this neighborhood is ____?” using concept mapping to engage communities as	Study (peer-reviewed).	Healthcare (includes paramedicine services), Social Services, Community Services, Government, and Research/Acad	Structurally Marginalized (e.g., disabled, elderly, unhoused, refugee, migrant, immigrant, incarcerated, etc.) Identity-	Urban setting: key informant sought from "priority populations": youth, seniors, individuals of Indigenous descent, immigrants,	Are there specific racial or identity considerations for the community? Outline stakeholders who should be involved with		Are specific vulnerabilities or characteristics associated with this population that may result in marginalization (e.g., older	What healthcare access points exist in the community, what services do they provide, and are they accessible and	What is the status of SDH for the community? Housing, Education, Public Transport, Access to Communication			Are social activities accessible within the community? Are social activities specifically aimed at adults over age 65?	No	Granularity of service availability as walk-in, after hours, 24/7, outreach for accessibility

Supplementary Information S1—Extracted Data

Author / Year	Country	Language	Document Title	Document Type(s)	Intended Audience(s)	Community Served (social unit)	Community Description	I. Community and stakeholders	II. Indigenous community considerations	III. Existing information	IV. Access to health and social care services	V. Daily living considerations	VI. Mental health and substance use	VII. Technology	VIII. Social support agency and belonging	Is intersectionality considered in the document?	Additional relevant content or context to include and consider in the development of a community needs assessment?
			part of a health and human services needs assessment.		emia.	based (e.g., race, ethnicity, gender, sexual orientation, religion, etc.). Geographical – Urban.	self-identified LGBT, Mandarin and Bengali speakers, and individuals experiencing mental health or addiction issues.	and/or consulted when conducting the community needs assessment.		adults/>65, minors, children in care, incarcerated, victims of human trafficking, sex workers, undocumented or illegal immigrants)? Is this community located in an urban, suburban, rural, mixed, isolated geographic setting, or bedroom community? Do language or communication considerations exist within the community? Existing information to gather on the community (data, as applicable).	equitable? What social care access points exist in the community, what services do they provide, and are they accessible and equitable? What social services exist in the community, what services do they provide, and are they accessible and equitable?	s (see Section VII), disability.			Are supports available to people experiencing the effects of violence or abuse within the community?		
2023 Summers-Gabr et al	United States	English	One Decade Later: The Generalizability , Diversity, and Inclusion of Community Health Needs Assessments	Study (peer-reviewed).	Healthcare (includes paramedicine services), Social Services, Community Services, Government, and Research/Academia.	Geographical – National.	study of generalizability and diversity of community health needs assessment data, from national data sources.									No	Data collected by hospitals and used to determine CHNA health priorities are not representative of the region they serve. ■ Well-defined regulations around data collection should be made so that community benefit dollars are invested into priorities that represent the whole population and not a subset of individuals. These regulations should include offering surveys in languages other than English, recruiting and offering verbal surveys for

Supplementary Information S1—Extracted Data

Author / Year	Country	Language	Document Title	Document Type(s)	Intended Audience(s)	Community Served (social unit)	Community Description	I. Community and stakeholders	II. Indigenous community considerations	III. Existing information	IV. Access to health and social care services	V. Daily living considerations	VI. Mental health and substance use	VII. Technology	VIII. Social support agency and belonging	Is intersectionality considered in the document?	Additional relevant content or context to include and consider in the development of a community needs assessment?
																	those who cannot read well, and recruiting and offering surveys in alternative ways for those who do not have Internet access. ■ The vast majority of hospitals do not collect any primary youth data when conducting a CHNA; youth participation should be incorporated to better understand and strategize how to improve health disparities for youth. ■ If the timeline requirements for hospitals' CHNA and public health departments' Community Health Assessment could be changed to the same length, the two entities could unify, collect better-quality data, and align improvement plans.
2017 Hernandez et al	United States	English	Oral Histories as Critical Qualitative Inquiry in Community Health Assessment	Study (peer-reviewed).	Healthcare (includes paramedicine services), and Research/Academia.	Structurally Marginalized (e.g., disabled, elderly, unhoused, refugee, migrant, immigrant, incarcerated, etc.). Identity-based (e.g., race, ethnicity, gender, sexual orientation, religion, etc.)	Urban, Mexican ethnic enclave.	Are there specific racial or identity considerations for the community?									Qualitative methods such as focus groups and interviews are common methodologies employed in participatory approaches to community health assessment to develop effective community health improvement plans. Oral histories are a rarely used form of qualitative inquiry that can enhance community health assessment in

Supplementary Information S1—Extracted Data

Author / Year	Country	Language	Document Title	Document Type(s)	Intended Audience(s)	Community Served (social unit)	Community Description	I. Community and stakeholders	II. Indigenous community considerations	III. Existing information	IV. Access to health and social care services	V. Daily living considerations	VI. Mental health and substance use	VII. Technology	VIII. Social support agency and belonging	Is intersectionality considered in the document?	Additional relevant content or context to include and consider in the development of a community needs assessment?
																	multiple ways. Oral histories center on residents' lived experiences, which often reveal more complex social and health phenomena than conventional qualitative inquiry. Oral histories were most meaningful in their original audio form, adding to a holistic understanding of health by giving voice to complex problems while also naming and describing concepts that were culturally unique. Moreover, the oral histories collectively articulated a counter-narrative that celebrated community cultural wealth and opposed the mainstream narrative of the community as deprived. We argue for the recognition and practice of oral histories as a more routine form of qualitative inquiry in community health assessment. In the pursuit of health equity and collaboratively working toward social justice, oral histories can push the boundaries of community health assessment research and practice.
2020 Santos	United States	English	Non-profit Hospital Targeted Health Priorities and Collaboration	Study (peer-reviewed).	Healthcare (includes paramedicine services), Social Services,	Structurally Marginalized (e.g., disabled, elderly, unhoused,		Are there specific racial or identity considerations for the		Is this community located in an urban, suburban, rural,		What is the status of SDH for the community? Income,	Are specific mental health concerns present within the			No	Inclusion of pediatric-specific services

Supplementary Information S1—Extracted Data

Author / Year	Country	Language	Document Title	Document Type(s)	Intended Audience(s)	Community Served (social unit)	Community Description	I. Community and stakeholders	II. Indigenous community considerations	III. Existing information	IV. Access to health and social care services	V. Daily living considerations	VI. Mental health and substance use	VII. Technology	VIII. Social support agency and belonging	Is intersectionality considered in the document?	Additional relevant content or context to include and consider in the development of a community needs assessment?
			With Local Health Departments in the First Round Post-ACA: A National Descriptive Study		Community Services, Research/Academia.	refugee, migrant, immigrant, incarcerated, etc.). Identity-based (e.g., race, ethnicity, gender, sexual orientation, religion, etc.). Geographical - Urban, Geographical - Rural, Geographical - Regional/County, Geographical - Provincial/State, and Geographical – National.		community?		mixed, isolated geographic setting, or bedroom community? Existing information to gather on the community (data, as applicable).		Employment, uninsured.	community? Is there a mental health facility accessible to the community (including inpatient services) availability of psychiatric beds?				
2022 Rayan-Gharra et al	Israel	English	Shaping health: conducting a community h ealth needs assessment in culturally diverse peripheral population groups.	Study (peer-reviewed).	Healthcare (includes paramedicine services), and Research/Academia.	Structurally Marginalized (e.g., disabled, elderly, unhoused, refugee, migrant, immigrant, incarcerated, etc.). Identity-based (e.g., race, ethnicity, gender, sexual orientation, religion, etc.). Geographical - Urban, Geographical - Rural, and Geographical - Regional/County.	Galilee residents, Israel	Are there specific racial or identity considerations for the community? Outline stakeholders who should be involved with and/or consulted when conducting the community needs assessment, including religion.		Is this community located in an urban, suburban, rural, mixed, isolated geographic setting, or bedroom community? Do language or communication considerations exist within the community? Existing information to gather on the community (data, as applicable).	What healthcare access points exist in the community, what services do they provide, and are they accessible and equitable? What social care access points exist in the community, what services do they provide, and are they accessible and equitable? What social services exist in the community, what services do they provide, and are they accessible and equitable? Race, ethnic discrimination impacting equitable access to services	What is the status of SDH for the community? Income, Housing, Food Security, Education, Employment, Public Transport, Infrastructure, Air Quality, and Drinking Water.	Are specific mental health concerns present within the community? Is there a mental health facility accessible to the community (including inpatient services)?		Are there specific cultural or ethnic considerations for the community? Are supports available to people experiencing the effects of violence or abuse within the community? Sense of community.	Yes, CHNA analyzed by ethnicity and municipal clusters.	Solutions: 1. improve the quality of care 2. community-based prevention partnerships 3. creating a regional integrated care model.
2023 Pazzaglia et al	Italy	English	Community Health Assessment Tools Adoptable in Nursing Practice: A Scoping Review	Scoping Review (peer-reviewed).	Healthcare (includes paramedicine services), and Research/Academia.	All of the above.	Scoping review of Community Health Needs Assessments.			Existing information to gather on the community (data, as applicable).		What is the status of SDH for the community?				No	Themes: education and skills of the health professional conducting the CNA; shared decision-making; community engagement and empowerment; "culturally competent"

Author / Year	Country	Language	Document Title	Document Type(s)	Intended Audience(s)	Community Served (social unit)	Community Description	I. Community and stakeholders	II. Indigenous community considerations	III. Existing information	IV. Access to health and social care services	V. Daily living considerations	VI. Mental health and substance use	VII. Technology	VIII. Social support agency and belonging	Is intersectionality considered in the document?	Additional relevant content or context to include and consider in the development of a community needs assessment?
																	approach; development of social policies; flexibility and adaptability of tools.
2008 Sharma	United States	English	Putting the Community Back in Community Health Assessment	Study (peer-reviewed).	Healthcare (includes paramedicine services), Social Services, Community Services, and Research/Academia.		Communities requiring a community health needs assessment (CHNA).										Definitions and dimensions of CHA (CHA as a continuum); defining and characterizing a community; defining health; 8-step process-focused conceptual model of a CHA.
2018 Akintobi et al	United States	English	Processes and Outcomes of a Community-Based Participatory Research-Driven Health Needs Assessment: A Tool for Moving Health Disparity Reporting to Evidence-Based Action	Study (peer-reviewed).	Healthcare (includes paramedicine services), and Research/Academia.		Communities requiring a community health needs assessment (CHNA).									No	Community-based participatory research-driven community health needs assessment (CBPR-driven CHNA).
2015 Schafer, Dawson	United States	English	Assessing Community Health Needs in a Rural Area: Determining Best Practices to Meet New Affordable Care Act Requirements	Study (peer-reviewed).	Healthcare (includes paramedicine services), and Research/Academia.	Geographical – Rural.	Rural communities requiring a community health needs assessment (CHNA).	Local school district.		Existing information to gather on the community (data, as applicable).						No	Develop community partnerships; use assortment of community-based participatory research (CBPR) data collection methods; data ownership and control - disseminate results broadly to community.
2016 Suiter	United States	English	Community health needs assessment and action planning in seven Dominican bateyes	Study (peer-reviewed).	Healthcare (includes paramedicine services), Social Services, Community Services, Research/Academia, and NGO.	Structurally Marginalized (e.g., disabled, elderly, unhoused, refugee, migrant, immigrant, incarcerated, etc.). Identity-based (e.g., race, ethnicity, gender, sexual orientation, religion, etc.), bateyes.	bateyes (impoverished communities in the Dominican Republic).	Are there specific racial or identity considerations for the community?		Are specific vulnerabilities or characteristics associated with this population that may result in marginalization (e.g., older adults/>65, minors, children in care, incarcerated, victims of human trafficking, sex workers, undocumented or illegal immigrants)? Is this community located in an		What is the status of SDH for the community?				Yes, access to health services; employment and health; environment and health; political power and health	Intersectional analysis; priority on child health.

Supplementary Information S1—Extracted Data

Author / Year	Country	Language	Document Title	Document Type(s)	Intended Audience(s)	Community Served (social unit)	Community Description	I. Community and stakeholders	II. Indigenous community considerations	III. Existing information	IV. Access to health and social care services	V. Daily living considerations	VI. Mental health and substance use	VII. Technology	VIII. Social support agency and belonging	Is intersectionality considered in the document?	Additional relevant content or context to include and consider in the development of a community needs assessment?
										urban, suburban, rural, mixed, isolated geographic setting, or bedroom community? Existing information to gather on the community (data, as applicable).							
2023 Ravaghi et al	United States	English	A scoping review of community health needs and assets assessment: concepts, rationale, tools, and uses	Scoping review.	Healthcare (includes paramedicine services), and Research/Academia.		Communities requiring a community needs assessment.									No	Terminology: Community Needs Assessment (CNA); Community Health Needs Assessment (CHNA); Community Health Needs and Asset Assessment (CHNAA); definitions of needs, differentiating between health need and healthcare need; definition of community; definition of assets; definition and key steps of a CHNAA
2023 Henrikson, Bishop	United States	English	Conducting a Community-Level Needs Assessment Through Dynamic Engagement With Stakeholders	Guideline, Study (peer-reviewed).	Healthcare (includes paramedicine services), and Research/Academia.	Geographical – Rural.	A K-12 school district in a rural area of Washington state.	Outline stakeholders who should be involved with and/or consulted when conducting the community needs assessment.		Is this community located in an urban, suburban, rural, mixed, isolated geographic setting, or bedroom community?	What social care access points exist in the community, what services do they provide, and are they accessible and equitable? What social services exist in the community, what services do they provide, are they accessible and equitable? Specifically assessed childcare needs of families with school-aged children.					No	strengths-based approach to cultivating partnerships; prioritized trust-building by applying community-based participatory research methods; phased approach to conducting CNA - Phase 1: The Strategic Development of Processes and Resources Phase 2: Refining the Focus for Deeper Insights Phase 3: The Comprehensive Analysis of Data
2018 Powell et al	United States	English	A Content Analysis of Nonprofit Hospital Community	Study (peer-reviewed).	Healthcare (includes paramedicine services), and Research/Acad	Geographical - Provincial/State .	Nonprofit hospital community health needs assessment									No	CHNA organized into domains: Clinical Care (access to care,

Supplementary Information S1—Extracted Data

Author / Year	Country	Language	Document Title	Document Type(s)	Intended Audience(s)	Community Served (social unit)	Community Description	I. Community and stakeholders	II. Indigenous community considerations	III. Existing information	IV. Access to health and social care services	V. Daily living considerations	VI. Mental health and substance use	VII. Technology	VIII. Social support agency and belonging	Is intersectionality considered in the document?	Additional relevant content or context to include and consider in the development of a community needs assessment?
			Health Needs Assessments and Community Benefit Implementation Strategies in Philadelphia		emia.		content analysis in Philadelphia.										quality of care), Health Behaviors, Physical Environment, and Social and Economic Factors.
2015 Pennel et al	United States	English	Nonprofit Hospitals' Approach to Community Health Needs Assessment	Study (peer-reviewed)	Healthcare (includes Public Health, paramedicine services), and Research/Academia	Geographical - Regional/County, Geographical - Provincial/State, prompted to define community.	Review of community health assessment and implementation strategy reports in Texas.	Outline stakeholders who should be involved with and/or consulted when conducting the community needs assessment.				What is the status of SDH for the community?				No	Examination of causation; identification and prioritization of needs; clear goals and measurable objectives.
2016 Santilli et al	United States	English	Applying Community Organizing Principles to Assess Health Needs in New Haven, Connecticut	Study (peer-reviewed)	Healthcare (includes Public Health, paramedicine services), and Research/Academia	Geographical - Regional/County.	New Haven, Connecticut.	Outline stakeholders who should be involved with and/or consulted when conducting the community needs assessment.		Existing information to gather on the community (data, as applicable).						No	Benefits of applying community-organizing principles to approaches for conducting CNA.
2020 Bias et al	United States	English	The Importance of Individual-Site and System-Wide Community Health Needs Assessments	Study (peer-reviewed).	Healthcare (includes Public Health, paramedicine services), and Research/Academia.	designated hospitals in determined medicine system.	Eight hospitals affiliated with West Virginia University medicine system.	Are there specific racial or identity considerations for the community? Outline stakeholders who should be involved with and/or consulted when conducting the community needs assessment.		Do language or communication considerations exist within the community? Existing information to gather on the community (data, as applicable).	What healthcare access points exist in the community, what services do they provide, and are they accessible and equitable? What social care access points exist in the community, what services do they provide, and are they accessible and equitable? What social services exist in the community, what services do they provide, and are they accessible and equitable?	What is the status of SDH for the community? Income, Housing, Food Security, Education, Employment, Public Transport, Infrastructure, Air Quality, and Drinking Water.	Are specific mental health concerns present within the community? Is there a mental health facility accessible to the community (including inpatient services)?		Are social activities accessible within the community?	No	
2001 Melton et al	United States	English	A Community Needs Assessment for a SANE Program Using Neuman's Model	Guideline, Study (peer-reviewed).	Healthcare (includes Public Health, paramedicine services), and Research/Academia	Structurally Marginalized (e.g., disabled, elderly, unhoused, refugee, migrant, immigrant, incarcerated, etc.). Identity-based (e.g., race, ethnicity, gender, sexual orientation,	Rape and sexual assault survivors.	Are there specific racial or identity considerations for the community? Outline stakeholders who should be involved with and/or consulted when conducting the community		Is this community located in an urban, suburban, rural, mixed, isolated geographic setting, or bedroom community? Do language or communication considerations exist within the	What social services exist in the community, what services do they provide, and are they accessible and equitable?	What is the status of SDH for the community? Income, Education, Employment, Public Transport, and Infrastructure	Are specific mental health concerns present within the community?			No	Guideline for a CNA for SANE Program using the Neuman Model that differentiates between intrapersonal environmental factors, interpersonal environmental factors, and extrapersonal

Supplementary Information S1—Extracted Data

Author / Year	Country	Language	Document Title	Document Type(s)	Intended Audience(s)	Community Served (social unit)	Community Description	I. Community and stakeholders	II. Indigenous community considerations	III. Existing information	IV. Access to health and social care services	V. Daily living considerations	VI. Mental health and substance use	VII. Technology	VIII. Social support agency and belonging	Is intersectionality considered in the document?	Additional relevant content or context to include and consider in the development of a community needs assessment?
						religion, etc.).		needs assessment.		community? Existing information to gather on the community (data, as applicable).							environmental factors. All include physiological, psychological, developmental, sociocultural, spiritual, and client perceptions; "client" is an open system.
2018 Van Gelderen et al	United States	English	Trialing the Community-Based Collaborative Action Research Framework: Supporting Rural Health Through a Community Health Needs Assessment	Guideline, Study (peer-reviewed).	Healthcare (includes Public Health, paramedicine services), and Research/Academia.		rural communities.									No	Uses the Community-Based Collaborative Action Research Framework (CBCAR) to facilitate community engagement and promote collective trust; developed by Pavlish and Pharris 2012
2017 Bias et al	United States	English	The Impact of Community Input in Community Health Needs Assessments	Study (peer-reviewed).	Healthcare (includes Public Health, paramedicine services), and Research/Academia.		Communities requiring a community needs assessment.	Outline stakeholders who should be involved with and/or consulted when conducting the community needs assessment, community participation and input is key to identifying unique health needs.									community input identified injury control and mental health as top priorities
2015 Pennel et al	United States	English	Community Health Needs Assessment: Potential for Population Health Improvement	Study (peer-reviewed).	Healthcare (includes Public Health, paramedicine services), Social Services, Community Services, and Research/Academia.		Communities requiring a community needs assessment.	Outline stakeholders who should be involved with and/or consulted when conducting the community needs assessment, defining community beyond hospital or clinic populations.				What is the status of SDH for the community? identifying broader determinants and points of intervention				No	recognizing population health improvement as a shared responsibility; identifying drivers and root causes of health issues; implementing clinical and non-clinical interventions
2018 Mannix et al							Utah urban American Indians and Alaskan Natives.	Veterans.	Are there Indigenous-specific barriers to healthcare utilization? Is there Elder care support services?	Existing information to gather on the community (data, as applicable).	What healthcare access points exist in the community, what services do they provide, and are they accessible and equitable? What social care access points exist in	What is the status of SDH for the community? Education, Employment, Disability.	Are specific mental health concerns present within the community? Is there a mental health facility accessible to the community (including inpatient services)?	What platforms or solutions are the community currently using? Are any community members disproportionately affected by a lack of access to technology? What barriers does the community face	Are social activities accessible within the community? Are there specific cultural or ethnic considerations for the community? classes - cooking, art, gardening	No	

Supplementary Information S1—Extracted Data

Author / Year	Country	Language	Document Title	Document Type(s)	Intended Audience(s)	Community Served (social unit)	Community Description	I. Community and stakeholders	II. Indigenous community considerations	III. Existing information	IV. Access to health and social care services	V. Daily living considerations	VI. Mental health and substance use	VII. Technology	VIII. Social support agency and belonging	Is intersectionality considered in the document?	Additional relevant content or context to include and consider in the development of a community needs assessment?
											the community, what services do they provide, and are they accessible and equitable? What social services exist in the community, what services do they provide, and are they accessible and equitable?			in using digital technology or tools? Are there technology assistance services?			
2017 Pennel et al	United States	English	A Mixed-Methods Approach to Understanding Community Participation in Community Health Needs Assessments	Study (peer-reviewed)	Healthcare (includes Public Health, paramedicine services), Social Services, Community Services, and Research/Academia.		Communities requiring a community needs assessment.	Outline stakeholders who should be involved with and/or consulted when conducting the community needs assessment; importance of community participation; emphasis on engaging a "broad array" of community partners when conducting CNA (e.g., non-health partners).								No	
2018 Cho et al	South Korea	English	Community health needs assessment: a nurses' global health project in Vietnam.	Study (peer-reviewed)	Healthcare (includes Public Health, Community Health, paramedicine services); Research/Academia.		Rural communities in Vietnam.	Outline stakeholders who should be involved with and/or consulted when conducting the community needs assessment.		Existing information to gather on the community (data, as applicable).	What healthcare access points exist in the community, what services do they provide, and are they accessible and equitable? What social care access points exist in the community, what services do they provide, and are they accessible and equitable? What social services exist in the community, what services do they provide, and are they accessible and equitable?					No	Public Health - access to immunizations; family planning; HIV services. Incorporate capacity of healthcare providers and capacity of community into CNA; wait times.

Supplementary Information S1—Extracted Data

Author / Year	Country	Language	Document Title	Document Type(s)	Intended Audience(s)	Community Served (social unit)	Community Description	I. Community and stakeholders	II. Indigenous community considerations	III. Existing information	IV. Access to health and social care services	V. Daily living considerations	VI. Mental health and substance use	VII. Technology	VIII. Social support agency and belonging	Is intersectionality considered in the document?	Additional relevant content or context to include and consider in the development of a community needs assessment?
2020 Berkley-Patton et al	Jamaica	English	Engaging Church Leaders in a Health Needs Assessment Process to Design a Multilevel Health Promotion Intervention in Low-resource Rural Jamaican Faith Communities	Study (peer-reviewed)	Healthcare (includes Public Health, Community Health, paramedicine services), Social Services, Community Services, Research/Academia	Identity-based (e.g., race, ethnicity, gender, sexual orientation, religion, etc.)	14 Jamaican faith parishes	Outline stakeholders who should be involved with and/or consulted when conducting the community needs assessment, involve faith communities in CNA, intervention design, and research process.								Yes	endorsed using community-based participatory research (CBPR) approaches to increase engagement with CNA, health promotion, and health services utilization
2016 Cain et al	United States	English	The Power of Community Voices for Enhancing Community Health Needs Assessments	Study (peer-reviewed)	Healthcare (includes Public Health, Community Health, paramedicine services), Social Services, Community Services, Research/Academia	Structurally Marginalized (e.g., disabled, elderly, unhoused, refugee, migrant, immigrant, incarcerated, etc.). Identity-based (e.g., race, ethnicity, gender, sexual orientation, religion, etc.). Geographical - Urban	Abbott Northwestern Hospital, Minneapolis, Minnesota	Are there specific racial or identity considerations for the community? Outline stakeholders who should be involved with and/or consulted when conducting the community needs assessment	View culture as a healthcare resource							Yes	Themes: view culture as a healthcare resource, foster community connections, be present
1994-2023 University of Kansas Centre for Community Health and Development	United States	English	Community Tool Box	Tool, free, online public service resource	Healthcare (includes Public Health, Community Health, paramedicine services), Education, Social Services, Community Services, Government, Research/Academia	All of the above	Communities requiring a community needs assessment	Outline stakeholders who should be involved with and/or consulted when conducting the community needs assessment		Existing information to gather on the community (data, as applicable), community history; social structure, governance structure	What healthcare access points exist in the community, what services do they provide, and are they accessible and equitable? What social care access points exist in the community, what services do they provide, and are they accessible and equitable? What social services exist in the community, what services do they provide, and are they accessible and equitable? Assess service utilization and service coordination	What is the status of SDH for the community?				No	Photovoice surveys; comprehensive preparatory checklists, facilitation guidance, analysis, and evaluation resources
